# Transcriptome datasets of oil palm pathogen *Ganoderma boninense*

**DOI:** 10.1016/j.dib.2018.02.027

**Published:** 2018-02-15

**Authors:** Irene Liza Isaac, A.W.C.Y. Walter, Mohd Faizal Abu Bakar, A.S. Idris, Farah Diba Abu Bakar, Izwan Bharudin, Abdul Munir Abdul Murad

**Affiliations:** a School of Biosciences and Biotechnology, Faculty of Science and Technology, Universiti Kebangsaan Malaysia, UKM, Bangi 43600, Selangor, Malaysia; b Malaysia Genome Institute, Ministry of Science, Technology and Innovation, Jalan Bangi, 43000 Kajang, Selangor, Malaysia; c Malaysian Palm Oil Board (MPOB), Persiaran Institusi, Bandar Baru Bangi, 43000 Kajang, Selangor, Malaysia

## Abstract

*Ganoderma boninense* is known to be the causal agent for basal stem rot (BSR) affecting the oil palm industry worldwide thus cumulating to high economic losses every year. Several reports have shown that a compatible monokaryon pair needs to mate; producing dikaryotic mycelia to initiate the infection towards the oil palm. However, the molecular events occurs during mating process are not well understood. We performed transcriptome sequencing using Illumina RNA-seq technology and *de novo* assembly of the transcripts from monokaryon, mating junction and dikaryon mycelia of *G. boninense*. Raw reads from these three libraries were deposited in the NCBI database with accession number SRR1745787, SRR1745773 and SRR1745777, respectively.

**Specifications table**

**Table:** 

Subject area	*Biology*
More specific subject area	*Molecular biology of fungal mating*
Type of data	*Transcriptome data*
How data was acquired	*Paired-end transcriptome of G. boninense was sequenced using Illumina HiSeq. 2000 at Malaysia Genome Institute. De novo transcriptome assembly was performed using Trinity V.2.0.2*
Data format	*Raw sequence (FASTQ)*
Experimental factors	*Mycelia from different stages; monokaryon, mating junction and dikaryon*
Experimental features	*G. boninense PER71 was obtained from Malaysian Palm Oil Board (MPOB). All cultures were maintained on Congo Red Yeast Mannitol Agar (CYMA).*
Data source location	*Bangi, Malaysia*
Data accessibility	*Raw FASTQ files were deposited in NCBI SRA database with accession number*SRR1745787, SRR1745773 and SRR1745777*(*https://www.ncbi.nlm.nih.gov/sra/SRX809922*)*

**Value of the data**

• The data obtained using Illumina sequencer is the first report on RNA-seq of *G. boninense* from three different stages; monokaryon, mating junction and dikaryon.
• The data presented here can be used for investigating the mating process in this fungus.
• This permits the identification of differentially expressed genes that may play a significant role in the pathogenesis of *G. boninense* towards oil palm.

## 1 Data

Transcriptome data of *Ganoderma boninense* were generated from the polyA-enriched cDNA libraries prepared from the total RNA extracted from three different stages as mentioned above. Details of the experimental procedure and sequence analyses were described in the next section.

## 2 Experimental design, materials and methods

### 2.1 Fungal strain, culture condition and inoculation

*G. boninense* PER71 dikaryon culture was obtained from Malaysian Palm Oil Board (MPOB). The fruiting body was grown and single spores were obtained which later are mated to obtain a compatible pair. All cultures were maintained on CYMA [maltose 1% (w/v), glucose 2% (w/v), yeast extract 0.2% (w/v), tryptone 0.2% (w/v), MgSO_4_.7H_2_O 0.05% (w/v), KH_2_PO_4_ (0.46%) (w/v)]. For transcriptome analysis, mycelia from monokaryon, mating interaction mycelia (mating junction) and dikaryon mycelia were grown on cellulose membrane placed on fresh CYMA plates which later left for 5 days at 30 °C before scrapping it out. As for mating interaction mycelia, a compatible monokaryon pair is used and maintained on CYMA individually. Agar disc of both monokaryon was dissected and placed within 2 cm between each other on a fresh CYMA plate. Plates were incubated at 30 °C for 4–5 days and the interacting mycelia are scrapped out. The obtained mycelia are frozen in liquid nitrogen and stored at −80 °C.

### 2.2 RNA extraction and sequencing

Total RNA from each of sample was isolated using RNeasy Plant Mini Kit (Qiagen, Germany) according to the manufacturer protocol. The integrity and quantity isolated RNA was quantified using Nanodrop and Bioanalyzer [1]. mRNA from each sample was isolated from 8 μg of total RNA using PolyATtract® mRNA Isolation Systems (Promega, USA) according to manufacturer's protocol. The cDNA library preparation was done using the Illumina mRNA Sequencing Sample Preparation (San Diego, CA, USA) as per the manufacturer's instructions. Libraries were built and quantified using a Qubit® 2.0 Fluorometer (Life Technologies, USA). Ten pM of the prepared library was loaded onto the flowcell for cluster generation as per instructions by cBot User Guide (Illumina, USA). Sequencing was performed with paired-end 2 × 100 bp and 2 × 93 bp nucleotides multiplex procedure on an Illumina HiSeq. 2000.

### 2.3 Assembly and RNA-seq analysis

The raw RNA-seq data from *G. boninense* was trimmed and filtered with SolexaQA v.2.2 [2] to acquire high-quality reads. Phred quality value of Q20 and reads longer than 50 bp were used as parameters. Paired-end reads were determined using Perl script *select_paired.pl. De novo* assembly of high-quality reads was carried out using Trinity V.2.0.2. [3], with default parameters. Table 1 shows the RNA-seq statistics whereas the assembly statistics as in Table 2.

**Table 1:** Statistics of the RNA-seq generated from three different libraries.

	Monokaryon	Mating junction	Dikaryon
Raw reads	94,798,526	103,341,138	131,501,350
Clean reads	82,999,421	85,822,868	114,260,189
Read counts for transcriptome assembly (paired-end reads)	76,459,032	76,887,432	104,993,372
Average read length (bp)	90	90	90
Total base pair (bp)	6,881,895,529	6,904,050,780	9,413,554,394

**Table 2:** Assembly statistics using Trinity.

Attributes	Value
Number of transcripts	35,903
Total residues (bp)	58,592,631
Average length (bp)	1632
N50 transcript	3118
Largest transcript (bp)	19,896
Smallest transcript (bp)	201
